# Methadone Maintenance Treatment Participant Retention and Behavioural Effectiveness in China: A Systematic Review and Meta-Analysis

**DOI:** 10.1371/journal.pone.0068906

**Published:** 2013-07-26

**Authors:** Lei Zhang, Eric P. F. Chow, Xun Zhuang, Yanxian Liang, Yafei Wang, Caiyun Tang, Li Ling, Joseph D. Tucker, David P. Wilson

**Affiliations:** 1 The Kirby Institute, University of New South Wales, Sydney, New South Wales, Australia,; 2 School of Public Health, Nantong University, Nantong, Jiangsu, China,; 3 Faculty of Medical Statistics and Epidemiology, School of Public Health, Sun Yat-sen University, Guangzhou, Guangdong, China; 4 Sun Yat-sen Center for Migrant Health Policy, Sun Yat-sen University, Guangzhou, Guangdong, China; 5 UNC Project-China, Guangzhou, Guangdong, China; 6 London School of Hygiene and Tropical Medicine, London, United Kingdom; Old Dominion University, United States of America

## Abstract

**Background:**

Methadone maintenance treatment (MMT) has been scaled up by the Chinese government alongside persistent compulsory drug user detention, but the extent to which detention interferes with MMT is unknown. The study systematically reviews Chinese MMT retention rates, reasons for drop out, and behavioural changes.

**Method:**

Chinese and English databases of literature are searched for studies reporting retention rates, drug use and sexual behaviours among MMT participants in China between 2004 and 2013. The estimates are summarized through a systematic review and meta-analysis.

**Results:**

A total of 74 studies representing 43,263 individuals are included in this analysis. About a third of MMT participants drop out during the first three months of treatment (retention rate 69.0% (95% CI 57.7-78.4%)). Police arrest and detention in compulsory rehabilitation was the most common cause of drop out, accounting for 22.2% of all those not retained. Among retained participants, changing unsafe drug use behaviours was more effective than changing unsafe sexual behaviours. At 12 months following MMT initiation, 24.6% (15.7-33.5%) of MMT participants had a positive urine test, 9.3% (4.7-17.8%) injected drugs and only 1.1% (0.4-3.0%) sold sex for drugs. These correspond to 0.002 (<0.001-0.011), 0.045 (0.004-0.114) and 0.209 (0.076-0.580) times lower odds than baseline. However, MMT participants did not have substantial changes in condom use rates.

**Conclusion:**

MMT is effective in drug users in China but participant retention is poor, substantially related to compulsory detention. Reforming the compulsory drug user detention system may improve MMT retention and effectiveness.

## Introduction

Since 1979 the illicit drug trade has prominently re-emerged in China [1], contributing to a substantial burden of drug-associated disease. The number of registered drug users in China increased 19-fold between 1990 and 2009 [1]. Intravenous injection is the most common means of drug use, with injecting drug users (IDUs) accounting for 59-85% of drug users in China [2,3,4,5,6,7,8]. The high injection frequency, sharing of contaminated needles and other risk behaviours [9,10,11] [12,13] among IDUs accelerate the spread of HIV infection. The cumulative number of diagnosed HIV/AIDS cases in China is now well over 200,000, among which over 60% are drug users [14,15], and new infections among IDUs accounts for 17% of new HIV cases in 2011 [16].

Responding to the growing IDU HIV epidemic in China, domestic and international programs launched harm reduction programs in 2003 [17,18,19]. A major component of harm reduction is methadone maintenance treatment (MMT), a substitution program known to reduce morbidity, a mortality [20,21] and risk of HIV infection in drug users [22]. China launched pilot MMT programs in 8 clinics serving 1,029 drug users in 2004 and subsequently expanded to 738 clinics serving 344,254 drug users by the end of 2011. Now MMT reaches approximately 30% of registered IDUs in China (personal communication with China CDC). MMT services focus on decreasing drug use and sexual risk behaviours, including counselling and HIV testing [23]. However, MMT services in China are closely monitored by the public security. Police raids and arrests near MMT sites are common, and MMT participants who have positive urine tests will be sent for compulsory detention and rehabilitation [23,24,25]. During and following detention and rehabilitation, access to MMT services is limited [24]. At places where the police is cooperative with the local CDC, participation and intervention outcomes in MMT participants are generally better [26].

While several studies have reported retention rates and behavioural outcomes in MMT participants [14,27,28,29,30,31,32,33,34,35,36,37,38,39,40,41,42,43,44,45,46,47,48,49,50,51,52,53,54,55, 56,57,58,59,60,61,62,63,64,65,66,67,68,69,70,71,72,73,74,75,76,77,78,79,80,81,82,83,84,85, 86,87,88,89,90,91,92,93,94,95,96,97,98,99], these studies have not been systematically reviewed and integrated to provide an overall assessment of the effectiveness of MMT programs in China [100,101]. Drop-out rates in MMT clinics are perceived to be high as approximately 50-70% participants terminate treatment within three month of their enrolment in China. Poor retention, administrative detention, and other structural factors may obscure the effect of MMT on durable behaviour change [102,103]. This study systematically investigates overall retention rates, reasons for dropout, drug use behaviour changes, and sexual behaviour changes at Chinese MMT clinics.

## Methods

### Search strategy

Two independent investigators (EPFC and XZ) conducted a systematic review of published peer-reviewed research articles by searching the following databases between January 2004 and April 2013: PubMed, Chinese Scientific Journals Fulltext Database (CQVIP), China National Knowledge Infrastructure (CNKI) and Wanfang Data. We performed two separate search strategies for (1) retention rates and reasons for dropout; and (2) behavioural changes attributable to MMT services. This review was reported according to the PRISMA (Preferred Reporting Items for Systematic Reviews and Meta-Analyses) Statement issued in 2009 [30] (Checklist S1). The search strategy was detailed in Supplementary Text S1.

### Study selection

Studies were eligible for inclusion in this systematic review if they met all following criteria: (1) study published in Chinese or English language; (2) study reported the drug and sexual behavioural changes before and after entering MMT clinics; (3) study reported percentage of retention rate among the MMT participants; (4) study reported reasons for dropout; and (5) study reported study site, time period and sample size. Intervention studies were selected, but only the control groups among MMT participants were included. Exclusion criteria were: (1) review papers; (2) non peer-reviewed local/government reports; (3) conference abstracts and presentations; (4) dissertations; (5) studies reported baseline or follow-up data only. If the same study data were published in both English and Chinese sources, the articles published in Chinese language journals were excluded from this study. A MMT participant was considered as ‘drop-out’ if he/she failed to attend the enrolled MMT clinic for seven consecutive days without providing a reason. All MMT clinics in China were under the supervision of China CDC and adapted the same definition of retention.

### Validity assessment

The quality of studies was assessed using a validated quality assessment tool [31]. The following eight items were assessed to calculate a total quality score: (1) clear definition of the target population; (2) representativeness of probability sampling; (3) sample characteristics matching the overall population; (4) adequate response rate; (5) standardised data collection methods; (6) reliability of survey measures/instruments; (7) validity of survey measures/instruments; (8) appropriate statistical methods. Answers were scored 0 and 1 for ‘No’ and ‘Yes’, respectively. The total quality score varied between 0 and 8 for each study.

### Data abstraction

We extracted the following information from all eligible studies: published year, study location, study period, age and sex composition of the sample, percentage married, level of education, study design, methods of recruitment and sample size at recruitment as the demographic indictors. We extracted the following behavioural change data for the pre-MMT and post-MMT periods: injecting drug in the past month, sharing needles in the past month, percentage who sell sex for drugs in the past three months, and the rate of consistent condom use during any sexual intercourse in the past month. Additionally, we extracted data on the retention rate of the MMT participants at several follow-up points, and the reasons for drop out. An individual was considered ‘dropped-out’ if they did not attend an MMT clinic for seven consecutive days.

### Statistical analysis

Meta-analyses were carried out with the Comprehensive Meta-Analysis software (V 2.0, Biostat, Englewood, New Jersey) [104]. The principal summary measures, including the effect rates of pooled prevalence estimates, odd ratios and their 95% confidence intervals (CIs) were determined based on random effect models. Random effect models were applied when heterogeneity across subgroups were found to be significant [105]. Heterogeneity tests were performed using the Cochran Q-test (*p*<0.10 represents statistically significant heterogeneity) and the *I* ^2^ statistic [106,107,108]. We investigated the factors that are associated with heterogeneities in the stratified meta-analyses using meta-regression [109]. Meta-regression was performed in STATA statistical software package (Version 10, StataCorp, College Station, TX). Potential publication bias was measured by the Begg and Mazumdar rank correlation (*p*<0.05) [110,111].

## Results

A total of 495 and 650 potentially relevant studies on MMT retention and behavioural changes, respectively, among drug users after MMT intervention were identified. A total of 56 [14,27,28,29,30,31,32,33,34,35,36,37,38,39,40,41,42,43,44,45,46,47,48,49,50,51,52,53,54,55, 56,57,58,59,60,61,62,63,64,65,66,67,68,69,70,71,72,73,74,75,76,77,78,79,80,81] (Figure S1) and 28 [30,34,37,44,49,50,62,63,64,81,82,83,84,85,86,87,88,89,90,91,92,93,94,95,96,97,98,99] studies (Figure S2) were subsequently eligible and selected for analyses, respectively.

### Retention in MMT

**Figure 1:**
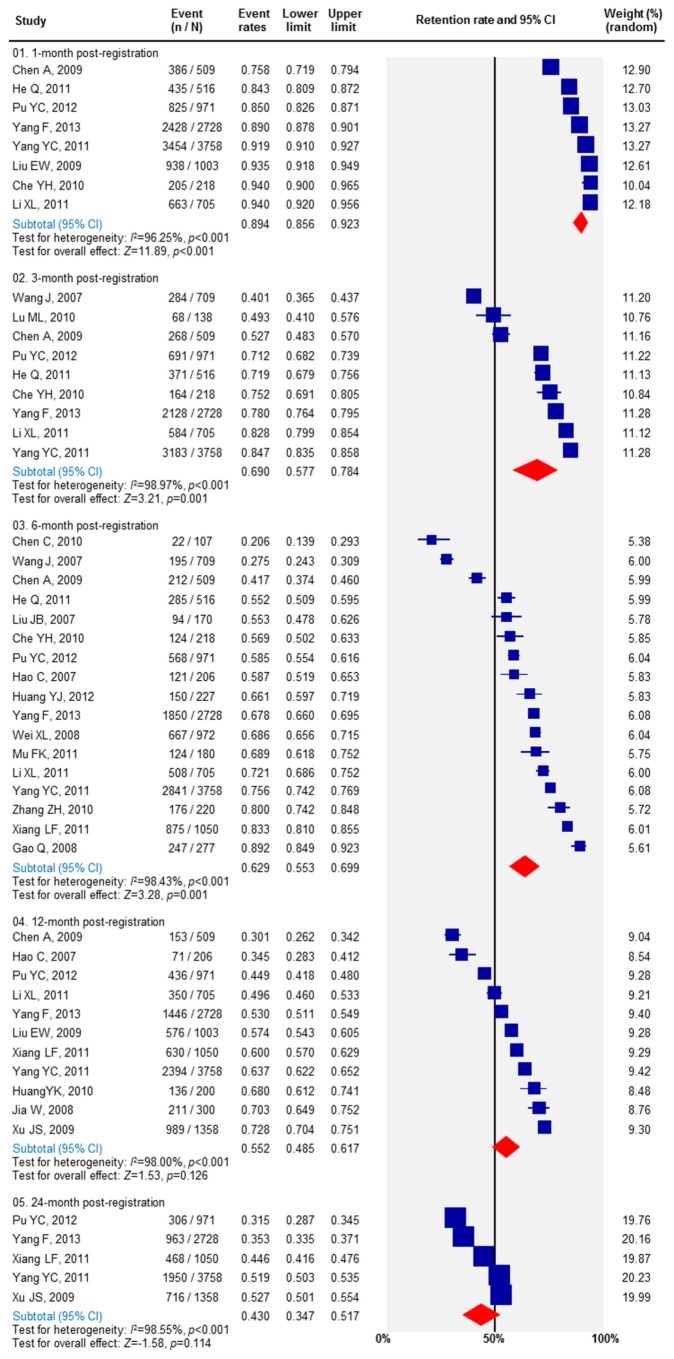
Retention of participants of MMT clinics in China.

**Figure 2:**
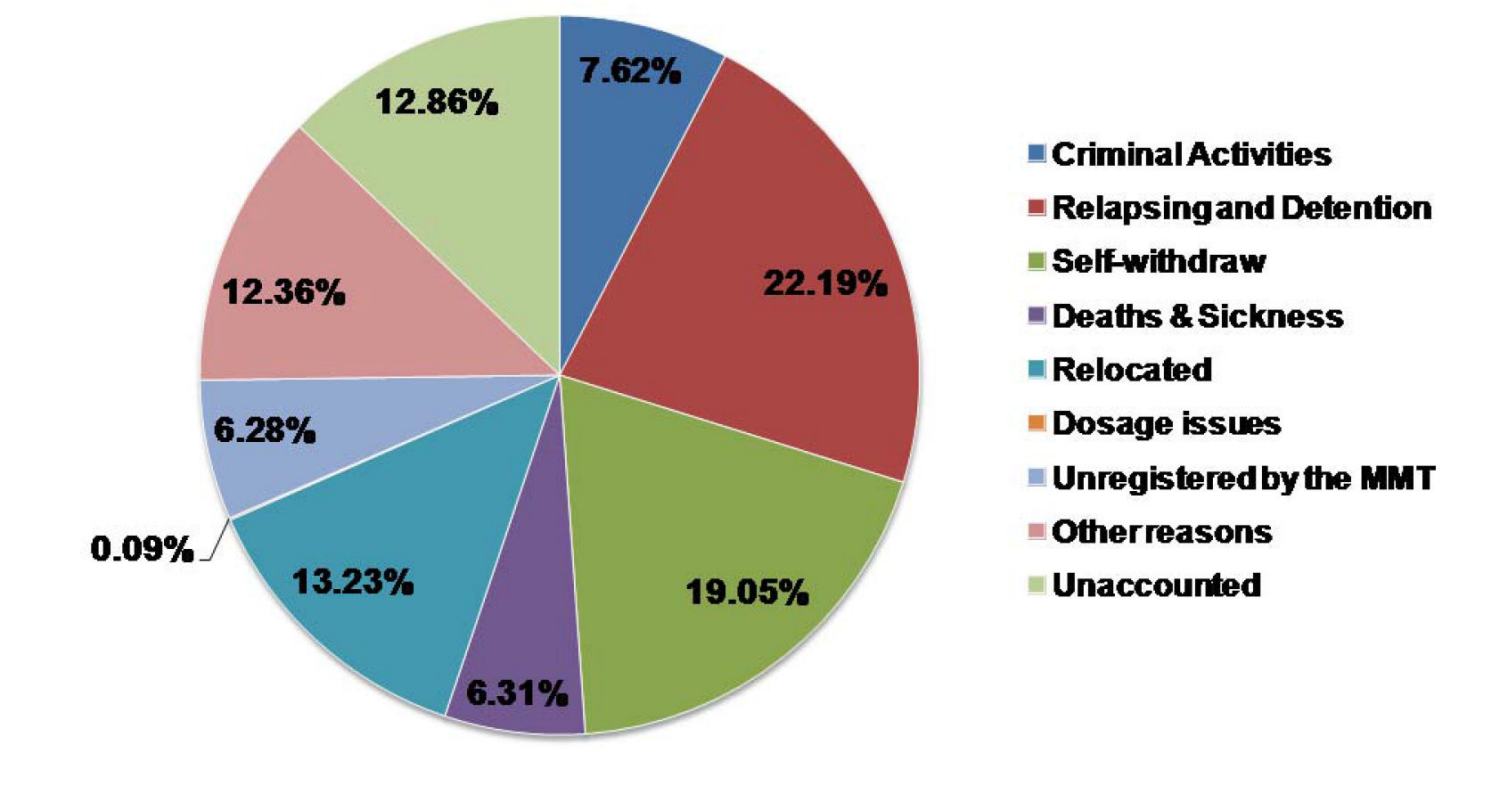
Major reasons of dropping-out from MMT clinics among 20,873 participants.

Twenty-seven studies reported retention rate among MMT participants (Table S1). The retention rates at one, three, six, 12, and 24 months after enrolment were 89.4% (95% CI, 85.6-92.3%), 69.0% (57.7-78.4%), 62.9 (55.3-69.9%), 55.2 (48.5-61.7%) and 43.0% (34.7-51.7%) (Figure 1). Notably, most drop outs occur in the first three months. MMT retention rates decrease less than 10% in the following 21 month period. Forty-three studies (20,873 studies participants) reported reasons for drop out from MMT in China (Table S2). Among the drop-outs, about one-fourth of the participants (22.2%) were arrested by police and sent to detention centres due to relapse in drug use (positive urine test) during the course of MMT, 19.1% self-withdrew and 13.3% were due to relocation to another city outside the clinic coverage. Other drop out reasons such as criminal activities (7.6%), unregistered by the MMT clinics (6.3%), death and sickness (6.3%) and dosage issues (0.1%) were also reported (Figure 2).

### Drug using behaviours among MMT participants

**Figure 3:**
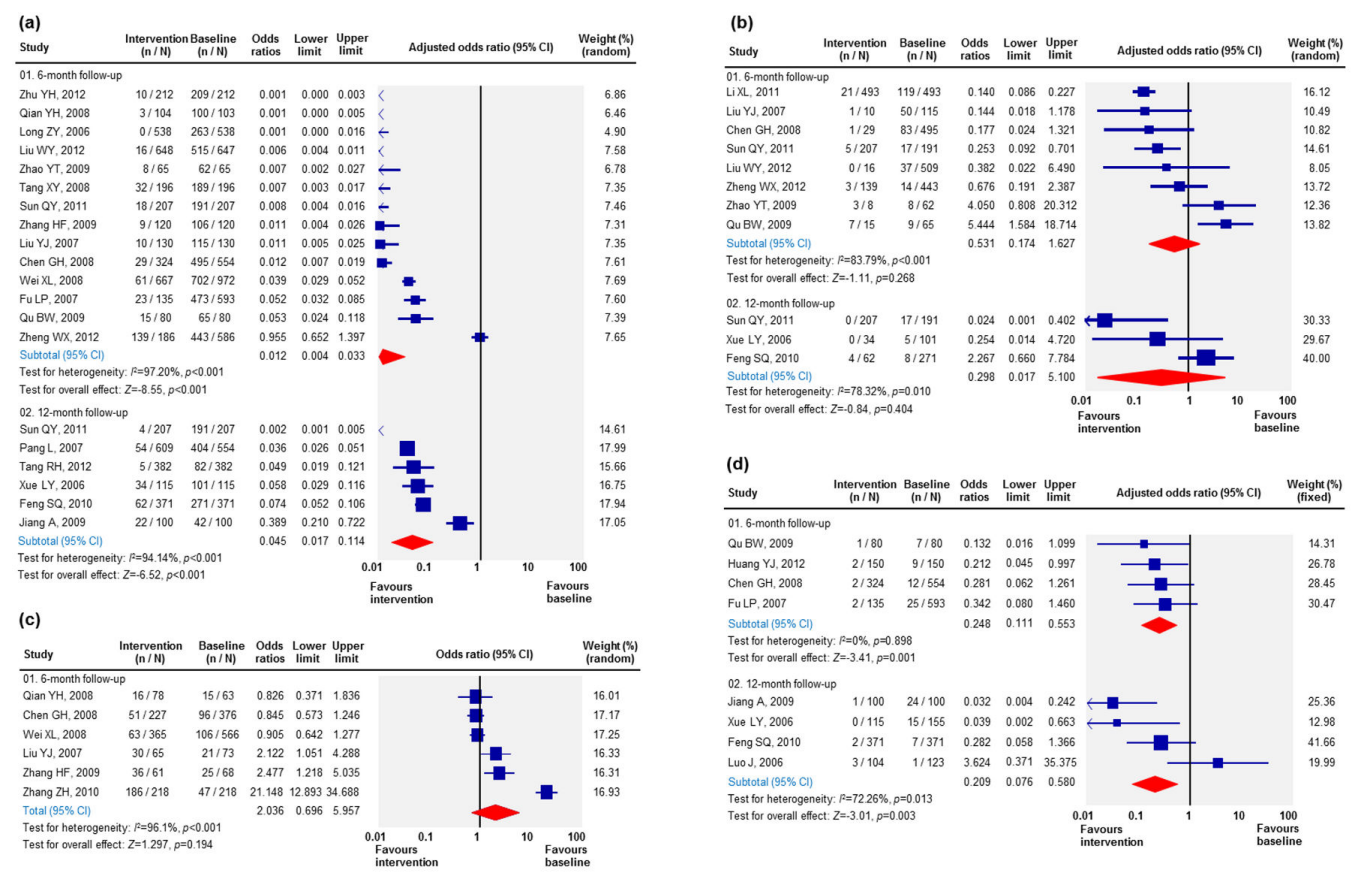
Changes in injecting behaviours among retained MMT participants. (a) Likelihood of injecting drug use in the past one month of Chinese MMT participants at six and twelve months follow-up. (b) Likelihood of sharing injection equipment in the past one month among Chinese MMT-participating IDUs at six and twelve months follow-up. (c) Likelihood of having consistent condom use during in all sexual intercourses in the past one month among Chinese MMT participants at 6 months follow-up. (d) Likelihood of selling sex for drugs in the past three months among Chinese MMT participants at six and twelve months follow-up.

Nineteen studies reported changes in injecting behaviours among MMT entrants after receiving MMT. Our analysis showed that 82.3% (75.2-87.7%) of the MMT entrants injected drugs in the past month at baseline and this significantly reduced to 9.1% (4.5-17.6%) after six months of entry and slightly increased to 9.3% (4.7-17.8%) after twelve months of MMT intervention (Figure S3a). These correspond to 0.012 (0.004-0.033) and 0.045 (0.017-0.114) times lower odds of injecting drug at six and twelve months after treatment respectively (Figure 3a). However, MMT did not change drug sharing behaviours among MMT participants (aOR = 0.531 [0.174-1.627] and 0.298 [0.017-5.100] for six and twelve months intervention) (Figure 3b). An estimated 10.8% (6.3-17.8%) of MMT participants shared syringes in the past month, whereas 7.6% (2.8-18.9%) and 1.8% (0.2-12.7%) of the MMT participants shared syringes after six and twelve months intervention (Figure S3b).

Six studies reported proportion of positive urine tests among MMT participants. At baseline, 92.6% (90.1-95.2%) of participants had positive drug urine tests. The proportion significantly dropped to 60.9% (53.5-68.3%), 50.8% (53.5-68.3%) and 24.6 (15.7-33.5%) at three, six and twelve months after enrolment in MMT (Table S3). These correspond to 0.47 (0.03-8.29), 0.05 (0.01-0.24) and 0.002 (<0.001-0.011) lower odds of positive urine testing compared with baseline.

### Sexual behaviours among MMT participants

Six studies reported the changes in sexual behaviours among MMT participants (Figure 3c). The consistent condom use in past one month increased from 24.6% (20.2-29.5%) at baseline level to 40.9% (18.7-67.6%) after 6 months of intervention, although the increase was not significant (aOR = 2.036 [0.696-5.957]) (Figure S3c).

Eight studies examined the likelihood of selling sex for drugs among MMT participants. Approximately 5.2% (2.5-10.3%) of MMT participants had sold sex for drugs in the past three months before receiving MMT intervention. This percentage dropped to 1.1% (0.5-2.3%) and 1.1% (0.4-3.0%) after six-month and 12-month post-intervention, respectively (Figure S3d). MMT had a significant impact on reducing commercial sex activities among drug users, as the odds of selling-sex in the past three months reduced to 0.248 (0.111-0.553) and 0.209 (0.076-0.580) six and 12 months after intervention (Figure 3d).

### Heterogeneities and publication biases

Heterogeneities were observed in several sub-group meta-analyses. In the meta-analyses of retention rate among MMT participants, high and significant heterogeneities were detected at 1 month (*I* ^2^=96.3%, *p*<0.001), 3 months (*I* ^2^=99.0%, *p*<0.001), 6 months (*I* ^2^=98.4%, *p*<0.001), 12 months (*I* ^2^=98.0%, *p*<0.001) and 24 months (*I* ^2^=98.6%, *p*<0.001) post-registration (Figure 1). Additionally, significant heterogeneities were also observed in measuring the OR of the percentage of who injected after six months (*I* ^2^=97.2%, *p*<0.001) and twelve months (*I* ^2^=94.1%, *p*<0.001) of follow-up (Figure 3a); the OR of the percentage who shared needles at six months follow-up (*I* ^2^=87.8%, *p*=0.002) (Figure 3b); the consistent condom use rate in the past one month (*I* ^2^=96.1%, *p*<0.001) after six-month follow-up (Figure 2c); and the OR of the percentage of MMT participants who sold sex for drug (*I* ^2^=72.3%, *p*=0.013) after twelve months follow-up (Figure 2d). Subsequent meta-regression suggested that sampling size, publication language, study design, period and location are not contributing factors to the observed high heterogeneities. No publications biases were observed in all meta-analyses performed. Of the full quality score of eight points, the selected studies have a median score of 4 (Interquartile range: 3-5) (Table S1-2, 4).

## Discussion

MMT is a key harm reduction strategy for improving the health and well-being of drug users worldwide. To our knowledge, this is the first systematic review of MMT effectiveness in a nation with common detention practices. Our analysis indicates that drop-out rates are high within the first three months of enrolment, as more than one-third of the participants discontinue their treatment. Retention increases substantially in the next 21 months, with over half of the participants remaining on treatment after 24 months. This demonstrates that participants who sustained the first three months are likely to remain on treatment. The phenomenon of early drop-out is consistent with findings in international settings [112,113]. In comparison, the 12-month MMT retention rate in China is substantially lower than programs in other developed (60-85% [22,114,115,116]) and developing countries settings (62-82% [117,118]).

The leading cause of MMT dropout (22.2%) was related to relapse in drug use and compulsory police detention. Our quantitative analysis extends previous qualitative literature suggesting interference between detention and MMT in Asia [102,103,119]. A recent 12 United Nation Joint Agency Statement called for an end to compulsory detention, as mandatory detention centres do not provide an effective environment for treatment of drug dependence and constantly violate internationally recognised human right standards [120].

Our results suggest that relapse and detention, self-withdrawal, and mobility are major barriers to durable MMT participant retention. The major reason for drop out is relapse and compulsory detention. In China, the police are entitled to request random urine tests for any suspected drug users. During times to meet arrest quotas, police will act aggressively toward drug users, especially those registered with MMT program [25]. Relapsed drug users are sent to detention centres. Detention centre confinement does not reduce unsafe drug use behaviours [121,122,123] and as many as 95% of IDUs relapse within one year of release from detention [124]. Access to general health care and harm reduction programs is extremely limited in Chinese detention centres [24]. Our results indicate that police arrest and forced detention of relapsing drug users may be a major obstacle for MMT program in China reaching its full potential in providing sustaining quality care for its participants. Second, more than one-fifth of the drop-outs choose to self-withdraw from the treatment. The self-perception of substantial improvement of physical conditions and alleviation of addictive symptoms during the early phase of MMT may lead to a misconception about maintaining drug abstinence without completing the course of treatment [125,126]. . The high self-withdraw highlights the need for improving necessary counselling services and peer support to eliminate these misconceptions [48,52,125]. Third, high mobility of MMT participants, reflected by their frequent relocations, also significantly contributes to the interruption of their treatment. This poses a great challenge to the current fragmented administrative model of MMT, in which MMT clinics in different administrative jurisdictions do not share the medical and treatment records of their participants [38,125,127,128]. Addressing these structural issues is important for retaining mobile drug users within the MMT system.

Our study found large reductions in drug-related risk behaviours among retained MMT participants in China. These findings are comparable to international research [129,130,131,132,133], indicating that the MMT has been effective in its core objective of reducing drug related risk. Nevertheless, these results need to be interpreted with caution. Individuals who relapse in China are more likely to be expelled from MMT and detained, decreasing their future chance of entering the MMT system. It may undermine the effectiveness of the program as relapsing individuals represent a subgroup with higher risks and needing the treatment the most. Notably, among continuing IDUs, the sharing rate of injection equipment did not change over the course of treatment, contradicting findings in other international contexts [129,130,131,134,135,136]. Further integration of MMT and syringe exchange programs is needed to reap the full benefits of harm reduction. The fact that MMT in China does not reduce unprotected sexual acts is consistent with international findings [137].

Several limitations in this study should be noted. First, our data covers only 19 provinces that are disproportionately in the south and south-western part of China. However, these regions have a larger number of IDUs with greater HIV disease burden. Although we systematically incorporated all available operational data, there remain a large number of governmental documents, community-level reports and other unpublished data that have never been archived in any of the public literature databases. For some indicators, the numbers of available publications are quite limited and this may potentially reduce the statistical power and accuracy of subsequent meta-analysis. Second, many studies report a high drop-out rate without indicating specific reasons (12.9% on average). Many contributing factors to treatment drop-out, such as attitudes of staff, under-dosing, costs of treatment, family support and commitments, stigma of being on methadone, poor psychosocial services, and ability for drop-out individuals to re-enter treatment, are not reported in the published literature and hence cannot be investigated. Third, only 0.1% of the drop-outs report insufficient methadone dosage as one of the reasons for leaving treatment [138]. International literature indicates that adequate methadone dosage should be above 60mg/day to be effective [128] and insufficient methadone dosage results in lower retention rates [138]. Daily dosage among Chinese MMT participants is substantially lower than this level and may have a strong effect on retention rates [38,125].

Our review provides pooled evidence that MMT has been effective in reducing drug-related risk behaviours among Chinese drug users. It informs policies to further expand the coverage and scope of MMT to provide better and more comprehensive treatment services for its participants. However, despite the 2008 revised Law on Drug Control allowing drug users in China access to community-based rehabilitation prior to compulsory detention [23], punitive incarceration of drug users in China remains common [139,140] and substantially impacts on participants’ retention. Our research adds quantitative public evidence for ending compulsory drug use detention, amplifying the recent UN joint statement [120] calling for an end to compulsory drug user detention. A recent pronouncement from the Chinese government stated that the re-education through labour system responsible for detaining drug users is likely to be reformed in the coming year [141]. Implementation of community-based rehabilitation in China and other states could substantially improve the effectiveness of MMT.

## Supporting Information

Table S1 Summary of the demographic characteristics of the studies reported retention rates in MMT clinics. Remove this caption text.
(DOCX)

Table S2 Major reasons of participants dropping-out from MMT clinics. Remove this caption text.
(DOCX)

Table S3 Percentage and likelihood of positive urine tests at baseline, 3, 6 and 12 months of follow-up. Remove this caption text.
(DOCX)

Table S4 Summary of the demographic characteristics of the studies that reported the changes in risk behaviours. Remove this caption text.
(DOCX)

Figure S1 Flow chart of study selection for retention among MMT participants. Remove this caption text.
(DOCX)

Figure S2 Flow chart of study selection for behavioural changes after MMT intervention. Remove this caption text.
(DOCX)

Figure S3 Changes in risk injecting and sexual behaviours among retained MMT participants. (a) Percentage of MMT participants who had injected drugs in the past one month. (b) Percentage of MMT participants who had shared syringe in the past one month. (c) Percentage of MMT participants who had consistent condom use during any sexual intercourse in the past one month. (d) Percentage of MMT participants who sell sex for drug in the past three months.
(DOCX)

Checklist S1 PRISMA Checklist. (DOCX)

Text S1 Search strategy. (DOCX)
