# Supplementary material for: Methadone Maintenance Treatment Participant Retention and Behavioural Effectiveness in China: A Systematic Review and Meta-Analysis
Source: PLoS One. 2013 Jul 26;8(7):e68906. doi: 10.1371/journal.pone.0068906 (PMC3724877; doi:10.1371/journal.pone.0068906)
Supplement: Table S1 — Remove this caption text. (DOCX) [file pone.0068906.s001.docx]

**Table S1. Summary of the demographic characteristics of the studies reported retention rates in MMT clinics.**

| **First author, published year** | **Language of Literature** | **Study Period** | **Study location** | **Province** | **Region** | **Type of Study** | **Method of sampling** | **Sampling size at recruitment** | **Age range (mean)** | **% of female** | **% of currently married** | **% of ethnic Han** | **Level of education (% Junior high and below)** | **% of internal migrants** | **Quality assessment score** |
| --- | --- | --- | --- | --- | --- | --- | --- | --- | --- | --- | --- | --- | --- | --- | --- |
| Che YH, 2010 [[1](#_ENREF_1)] | English | 2008/03-2009/02 | Kunming city | Yunnan | Southwest | Cohort | - | 218 | N/A (33.3) | 15.1 | 58.7% | 74.8% | 83.5% | - | 4 |
| Chen A, 2009  [[2](#_ENREF_2)] | Chinese | 2006/01-2008/10 | Taishan city, Yangdong county | Guangdong | South Central | Cohort | - | 509 | N/A (32.6) | 4.7% | 41.1% | - | 84.5% | - | 5 |
| Chen C, 2010 [[3](#_ENREF_3)] | Chinese | 2008 | Huaihua city | Hunan | South Central | Cohort | - | 107 | 15-59 (32.5) | 17.8% | - | - | - | - | 3 |
| Chen GH, 2008 [[4](#_ENREF_4)] | Chinese | 2006/02-2006/11 | Nanjing, Zhenjiang, Wuxi city | Jiangsu | East | Before-and-after | - | 554 | 13-52 (25.01) | 21.7% | 39.2% | 98.2% | 69.1% | - | 3 |
| Gao Q, 2008 [[5](#_ENREF_5)] | Chinese | 2007/03-2007/06 | Wuxi city | Jiangsu | East | Cohort | Convenience sampling | 277 | N/A (36.78) | 19.5% | 46.6% | 100.0% | 91.6% | - | 4 |
| Hao C, 2007 [[6](#_ENREF_6)] | Chinese | 2004/03-2005/10 | Xichang city | Sichuan | Southwest | Cohort | - | 206 | 21-53 (32.9) | 30.6% | 39.8% | 82.5% | 71.4% | - | 5 |
| He Q, 2011 [[7](#_ENREF_7)] | English | 2006/01-2009/05 | Taishan city, Yangdong county | Guangdong | South Central | Cohort | - | 516 | - | 4.7% | 38.4% | - | 85.3% | - | 5 |
| Huang YJ, 2012 [[8](#_ENREF_8)] | Chinese | 2009/10-2010/12 | Baise | Guangxi | South Central | Before-and-after | - | 227 | N/A(34.2) | 17.3% | 28.7% | 24.7% | 81.3% | - | 5 |
| Huang YK, 2010 [[9](#_ENREF_9)] | Chinese | 2005-2006 | Liuzhou city | Guangxi | South Central | Before-and-after | Random sampling | 450 | N/A (35) | 21.0% | - | - | - | - | 3 |
| Jia W, 2008 [[10](#_ENREF_10)] | Chinese | 2005/10-2006/01 | Xi'an city | Shaanxi | Northwest | Cohort | Stratified sampling | 300 | 21-51 (36.5) | 15.3% | - | - | - | - | 5 |
| Li XL, 2011 [[11](#_ENREF_11)] | Chinese | 2005/09-2007/04 | Hengyang, Yueyang, Changsha, Hongjiang, Chenzhou, Qidong, Shaodong | Hunan | South Central | Before-and-after | - | 705 | N/A | 15.0% | 15.0% |  |  | - | 3 |
| Liu E, 2009 [[12](#_ENREF_12)] | English | 2006/06-2007/06 | Anshun, Zunyi, Tongen, Shengeryi, Yuneryi, Duyu, Jianyu, Panxian city | Guizhou | Southwest | Cohort | - | 1,003 | - | 26.0% | 34.9% | 88.6% | - | 5.8% | 5 |
| Liu JB,2007 [[13](#_ENREF_13)] | Chinese | 2005/08-2005/10 | Ürümchi | Xinjiang | Northwest | Cohort | - | 170 | N/A (33.86) | 15.3% | 63.5% | 57.6% | - | - | 4 |
| Lu ML, 2010 [[14](#_ENREF_14)] | Chinese | 2007/02-2009/08 | Linxiang City | Hunan | South Central | Cohort | Stratified sampling | 138 | - | 18.8% | 52.9% | - | 74.6% | - | 3 |
| Mu FK, 2011 [[15](#_ENREF_15)] | Chinese | 2007/09-2010/06 | Guangyuan | Sichuan | Southwest | Cohort | - | 180 | N/A (37.5) | 25.6% | 43.9% | - | - | - | 4 |
| Pu YC, 2012 [[16](#_ENREF_16)] | Chinese | 2005/12-2011/06 | Longchuan | Yunnan | Southwest | Cohort | - | 971 | - | 3.5% | 66.6% | - | 58.4% | - | 4 |
| Wang J, 2007 [[17](#_ENREF_17)] | Chinese | 2005/08-2006/02 | Ürümchi | Xinjiang | Northwest | Cohort |  | 709 | - | 12.8% | 60.3% | 41.6% | 59.1% | - | 5 |
| Wei XL, 2008 [[18](#_ENREF_18)] | Chinese | 2005/10-2007/09 | Xi'an city | Shaanxi | Northwest | Before-and-after | Random sampling | 972 | N/A (37.08) | 16.0% | 51.0% | 90.7% | 63.3% | - | 5 |
| Xiang LF, 2011 [[19](#_ENREF_19)] | Chinese | 2005/06-2006/05 | Dehong | Yunnan | Southwest | Cohort Study | - | 1050 | NA(36.61) | 4.5% | 62.8% | 44.3% | 84.8% | - | 4 |
| Xu JS, 2009 [[20](#_ENREF_20)] | Chinese | 2006/02-2008/06 | - | Jiangsu | East | Cohort | - | 1,358 | 21-65 (36) | 22.8% | - | - | - | - | 3 |
| Yang F, 2013 [[21](#_ENREF_21)] | English | 2006/01-2010/09 | - | Guangdong | South Central | Cohort | Random sampling | 2728 | - | 27.2% | 51.3% | - | 79.9% | - | 5 |
| Yang YC, 2011 [[22](#_ENREF_22)] | Chinese | 2005-2009 | Dehong prefecture | Yunnan | Southwest | Cohort | Census | 3,758 | - | 4.2% | 55.5% | 43.0% | 86.6% | - | 5 |
| Zhang ZH, 2010 [[23](#_ENREF_23)] | Chinese | 2008/12-2009/12 | Huarong county | Hunan | South Central | Before-and-after | - | 220 | - | 15.5% | 46.4% | - | 28.7% | - | 2 |
| Zhao YT, 2009 [[24](#_ENREF_24)] | Chinese | 2006 | Guangzhou city | Guangdong | South Central | Before-and-after | - | 65 | N/A (36.75) | 6.2% | - | 100.0% | 78.5% | - | 6 |

**References**

1. Che Y, Assanangkornchai S, McNeil E, Chongsuvivatwong V, Li J, et al. (2010) Predictors of early dropout in methadone maintenance treatment program in Yunnan province, China. Drug Alcohol Rev 29: 263-270.

2. Chen A (2009) Predictors of retention related factors at the initial methadone maintenance treatment clinics in Guangdong province. Chinese Journal of Epidemiology 30: 1230-1233.

3. Chen C (2010) Analysis of hydrochloric acid Methadone Maintenance Treatment for Heroin Addicts. Chinese Community Doctors 12: 142-143.

4. Chen GH, Yang HT, Qian XC, Xu GY, Zhu YF, et al. (2008) Effectiveness evaluation of six-month community-based methadone maintenance treatment in Jiangsu Province. Chinese Journal of AIDS & STD: 590-593.

5. Gao Q, Cheng H, Ding L, Qian XC, Wang B (2008) Study on characteristics of social network among methadone maintenance treatment patients and retention situation. Acta Universitatis Medicinalis Nanjing (Social Science) 8: 230-233.

6. Hao C, Wu JL, Ruan YH, Yao HM, Yang XG, et al. (2007) Factors associated with retention in a community-based methadone maintenance treatment among heroin addicts. Zhonghua Yu Fang Yi Xue Za Zhi 41: 250-253.

7. He Q, Wang X, Xia Y, Mandel JS, Chen A, et al. (2011) New community-based methadone maintenance treatment programs in Guangdong, China, and their impact on patient quality of life. Subst Use Misuse 46: 749-757.

8. Huang YJ, Huang JG, Lv K, Zhou MX, Xia CL, et al. (2012) An outcome assessment on methadone maintenance treatment in Youjiang district, Baise city. Journal of Youjiang Medical University for Nationalities 34: 461-463.

9. Huang YK, Hu JL, Huang JP, Feng XX, Kong HQ (2010) Effect on psychological and behavior intervention among drug users in methadone maintenance treatment. Jilin Medical Journal 31: 153-154.

10. Jia W, Ye ZS, Zhang R, Wang RQ (2008) Analysis of dose-effect relationship and compliance for methadone maintenance treatment. Chinese Journal of Drug Abuse Prevention and Treatment 14: 253-256.

11. Li XL, Tan HZ, Ou QY, Chen MS, Zhang H (2011) Efficacy of methadone maintenance treatment among HIV positive and HIV negative heroin addicts. Chinese Journal of Drug Dependence 20: 362-366.

12. Wang P, Zhu JH, Yan J, Ou YS, Liu CF, et al. (2009) Analysis of patients' dropout from methadone maintenance treatment. Chinese Journal of Drug Abuse Prevention and Treatment 15: 77-80.

13. Liu JB, Di LXTHP, Li F, Zhang F, Mo LR, et al. (2007) The Effective Evaluation of the Methadone Maintenance Treatment of Heroin Addicts. Chinese Journal of Drug Abuse Prevention and Treatment 13: 10-13.

14. Lu ML, Li XG, Hu LY, Li F, Luo LH (2010) Influencing factors for dropout of 138 drug users on methadone maintenance treatment. Practical Preventive Medicine 17: 171-172.

15. Mu FK (2011) A study on 180 drug addicts on methadone maintenance treatment. China Medicine and Pharmacy 01: 117-118.

16. Pu YC, Long YC, Duan QX, Mo HP, Lu ZQ, et al. (2012) Adherence and determinants of methadone maintenance treatment among heroin addicts in Longchuan County,Yunnan Province. Chinese Journal of AIDS & STD 18: 815-817.

17. Wang J, Li F, Mo LR, Li L, Guan YS, et al. (2007) Factors associated with retention in a community-based methadone maintenance treatment among drug users in Urumqi, Xinjiang Uigur Autonomous Region. Zhonghua Liu Xing Bing Xue Za Zhi 28: 37-41.

18. Wei XL, Li HX, Ma CF, Liu JF (2008) Effective evaluation of methadone maintenance treatment for heroin dependent patients in Xi'an city. Chinese Journal of Drug Dependence 17: 197-201.

19. Xiang LF, Liu P, Gao J, Yang YC, Ye RH, et al. (2011) Evaluation of effectiveness of methadone maintenance treatment in Dehong prefecture of Yunnan. Chinese Journal of AIDS & STD 17: 426-429.

20. Xu JS, Chen GH, Li L, Huan XP, Yang HT, et al. (2009) Retention and related factors of methadone treatment among drug users. Zhonghua Liu Xing Bing Xue Za Zhi 25: 779-780.

21. Yang F, Lin P, Li Y, He Q, Long Q, et al. (2013) Predictors of retention in community-based methadone maintenance treatment program in Pearl River Delta, China. Harm Reduct J 10: 3.

22. Yang YC, Duan S, Xiang LF, Ye RH, Gao J, et al. (2011) Adherence and related determinants on methadone maintenance treatment among heroin addicts in Dehong prefecture, Yunnan province. Zhonghua Liu Xing Bing Xue Za Zhi 32: 125-129.

23. Zhang ZH, Yang NB (2010) Efficacy Evaluation of Methadone Maintenance Therapy on Drug Users in Huarong Country from 2008 to 2009. Practical Preventive Medicine 17: 1884-1886.

24. Zhao YT, Xu HF, Fan LR (2009) Evaluation for the Community-Based Methadone Maintenance Treatment in Guangzhou City. Journal of Tropical Medicine 9: 329-331.
