# Supplementary material for: Methadone Maintenance Treatment Participant Retention and Behavioural Effectiveness in China: A Systematic Review and Meta-Analysis
Source: PLoS One. 2013 Jul 26;8(7):e68906. doi: 10.1371/journal.pone.0068906 (PMC3724877; doi:10.1371/journal.pone.0068906)
Supplement: Table S2 — Remove this caption text. (DOCX) [file pone.0068906.s002.docx]

**Table S2. Major reasons of participants dropping-out from MMT clinics**

| **First author, published year** | **Number of drug users dropped out during intervention** | **Reasons of dropped out** | | | | | | | | | | | | | | | | | | **Quality assessment score** |
| --- | --- | --- | --- | --- | --- | --- | --- | --- | --- | --- | --- | --- | --- | --- | --- | --- | --- | --- | --- | --- |
|  |  | **Drug-related Crimes** | | **Relapsing and Detention** | | **Self-withdraw** | | **Deaths & Sickness** | | **Relocation** | | **Dosage issues** | | **Unregistered by MMT** | | **Other reasons** | | **Unaccounted** | |  |
|  |  | n | % | n | % | n | % | n | % | n | % | n | % | n | % | n | % | n | % |  |
| Cao XY, 2009 [[1](#_ENREF_1)] | 61 | 18 | 29.5% |  |  | 16 | 26.2% | 2 | 3.3% | 4 | 6.6% |  |  |  |  | 21 | 34.5% |  |  | 3 |
| Chen A, 2007 [[2](#_ENREF_2)] | 388 |  |  | 93 | 24.0% | 144 | 37.1% | 11 | 2.8% | 98 | 25.3% |  |  |  |  | 42 | 10.8% |  |  | 5 |
| Chen C, 2010 [[3](#_ENREF_3)] | 85 | 10 | 11.8% | 59 | 69.4% |  |  |  |  |  |  |  |  |  |  |  |  | 16 | 18.8% | 3 |
| Chen GH, 2008 [[4](#_ENREF_4)] | 42 | 24 | 57.1% |  |  | 2 | 4.8% | 1 | 2.4% | 7 | 16.7% |  |  |  |  |  |  | 8 | 19.0% | 3 |
| Chen W, 2010 [[5](#_ENREF_5)] | 7,732 |  |  | 1,701 | 22.0% | 2,015 | 26.1% | 236 | 3.1% | 830 | 10.7% |  |  | 50 | 0.7% | 1,240 | 16.0% | 1,660 | 21.5% | 4 |
| Cheng XL, 2009 [[6](#_ENREF_6)] | 76 | 10 | 13.2% | 15 | 19.8% | 6 | 7.9% | 7 | 9.2% | 38 | 50.0% |  |  |  |  |  |  |  |  | 4 |
| CuiZL,2012 [[7](#_ENREF_7)] | 112 | 4 | 3.6% | 8 | 7.1% | 4 | 3.6% | 8 | 7.1% | 81 | 72.3% |  |  |  |  | 7 | 6.3% |  |  | 4 |
| Feng WD, 2007 [[8](#_ENREF_8)] | 173 |  |  | 38 | 22.0% | 23 | 13.3% | 11 | 6.4% | 101 | 58.4% |  |  |  |  |  |  |  |  | 4 |
| Hao C, 2007 [[9](#_ENREF_9)] | 85 | 7 | 8.2% | 23 | 27.1% | 5 | 5.9% | 3 | 3.5% | 27 | 31.8% |  |  |  |  | 3 | 3.5% | 17 | 20.0% | 5 |
| He Q, 2011 [[10](#_ENREF_10)] | 231 |  |  | 70 | 30.3% | 56 | 24.4% | 14 | 6.1% | 91 | 39.3% |  |  |  |  |  |  |  |  | 5 |
| Huang YM, 2012 [[11](#_ENREF_11)] | 1256 |  |  | 85 | 6.8% | 86 | 6.8% | 241 | 19.2% | 165 | 13.1% |  |  | 512 | 40.8% | 167 | 13.3% |  |  | 3 |
| Jia W, 2008 [[12](#_ENREF_12)] | 89 |  |  | 15 | 16.9% |  |  | 15 | 16.9% | 27 | 30.3% | 5 | 5.6% |  |  | 27 | 30.3% |  |  | 5 |
| Jiang JH, 2011 [[13](#_ENREF_13)] | 290 | 136 | 46.9% | 30 | 10.3% | 56 | 19.3% | 56 | 19.3% | 4 | 1.4% |  |  |  |  | 8 | 2.8% |  |  | 3 |
| KongHQ,2011 [[14](#_ENREF_14)] | 311 |  |  | 111 | 35.7% | 9 | 2.9% | 130 | 41.8% |  |  |  |  |  |  | 52 | 16.7% | 9 | 2.9% | 4 |
| Lai WS, 2007 [[15](#_ENREF_15)] | 220 | 12 | 5.4% | 73 | 33.3% | 32 | 14.5% | 6 | 2.7% |  |  |  |  |  |  | 97 | 44.1% |  |  | 2 |
| Li XM, 2011 [[16](#_ENREF_16)] | 77 | 4 | 5.2% | 30 | 39.0% | 18 | 23.4% | 5 | 6.5% | 7 | 9.1% |  |  | 2 | 2.6% |  |  | 11 | 14.3% | 4 |
| Liu JB, 2007 [[17](#_ENREF_17)] | 36 | 6 | 16.7% |  |  | 11 | 30.6% |  |  | 5 | 13.9% |  |  |  |  | 3 | 8.3% | 11 | 30.6% | 4 |
| Liu YJ, 2007 [[18](#_ENREF_18)] | 73 | 27 | 37.0% |  |  |  |  | 4 | 5.5% | 32 | 43.8% |  |  |  |  | 10 | 13.7% |  |  | 3 |
| Lu ML, 2010 [[19](#_ENREF_19)] | 80 | 10 | 12.5% | 33 | 41.3% |  |  |  |  | 33 | 41.3% |  |  |  |  | 1 | 1.2% | 3 | 3.7% | 3 |
| Mu FK, 2011 [[20](#_ENREF_20)] | 56 | 43 | 76.8% |  |  |  |  |  |  |  |  |  |  | 13 | 23.2% |  |  |  |  | 4 |
| Pang L, 2007 [[21](#_ENREF_21)] | 1,567 | 92 | 5.8% | 483 | 30.8% |  |  |  |  | 220 | 14.0% |  |  | 212 | 13.6% |  |  | 560 | 35.8% | 5 |
| Qian YH, 2008 [[22](#_ENREF_22)] | 61 | 32 | 52.5% |  |  | 10 | 16.4% |  |  |  |  | 1 | 1.6% | 1 | 1.6% | 17 | 27.9% |  |  | 4 |
| Shuai YL, 2010 [[23](#_ENREF_23)] | 511 | 174 | 34.0% | 31 | 6.1% | 172 | 33.7% | 4 | 0.8% | 17 | 3.4% |  |  |  |  | 16 | 3.1% | 97 | 19.0% | 3 |
| Wang B, 2010 [[24](#_ENREF_24)] | 467 |  |  | 343 | 73.4% | 124 | 26.6% |  |  |  |  |  |  |  |  |  |  |  |  | 3 |
| Wang CQ, 2009 [[25](#_ENREF_25)] | 317 |  |  | 58 | 18.3% |  |  |  |  |  |  |  |  | 259 | 81.7% |  |  |  |  | 4 |
| Wang P, 2009 [[26](#_ENREF_26)] | 148 | 67 | 45.0% | 32 | 21.5% | 5 | 3.4% | 17 | 11.4% | 10 | 6.7% |  |  |  |  | 3 | 2.0% | 15 | 10.1% | 3 |
| Wang YZ, 2008 [[27](#_ENREF_27)] | 72 | 6 | 8.3% | 45 | 62.5% | 8 | 11.1% | 3 | 4.2% | 9 | 12.5% |  |  |  |  | 1 | 1.4% |  |  | 3 |
| Wang ZC, 2007 [[17](#_ENREF_17)] | 42 | 12 | 28.6% | 10 | 23.8% |  |  |  |  | 10 | 23.8% |  |  |  |  | 10 | 23.8% |  |  | 4 |
| Xing XZ, 2012 [[28](#_ENREF_28)] | 222 |  |  | 52 | 23.4% | 78 | 35.1% | 4 | 1.8% | 31 | 14.0% |  |  | 18 | 8.1% | 35 | 15.8% | 4 | 1.8% | 4 |
| Xu JS, 2009 [[29](#_ENREF_29)] | 590 | 197 | 33.4% | 110 | 18.6% | 104 | 17.6% | 38 | 6.4% | 48 | 8.1% | 3 | 0.5% | 49 | 8.3% | 6 | 1.0% | 35 | 5.9% | 3 |
| Xue LY, 2008 [[30](#_ENREF_30)] | 403 | 29 | 7.2% | 118 | 29.3% | 67 | 16.6% | 30 | 7.5% | 88 | 21.9% |  |  | 24 | 6.0% | 9 | 2.2% | 38 | 9.4% | 3 |
| Yan JF, 2010 [[31](#_ENREF_31)] | 107 |  |  | 27 | 25.2% | 43 | 40.2% | 6 | 5.6% |  |  |  |  |  |  | 31 | 28.9% |  |  | 2 |
| Yan L, 2009 [[32](#_ENREF_32)] | 250 | 5 | 2.0% | 19 | 7.6% | 43 | 17.2% | 20 | 8.0% | 100 | 40.0% | 6 | 2.4% | 15 | 6.0% | 2 | 0.8% | 40 | 16.0% | 4 |
| Yang LS, 2008 [[33](#_ENREF_33)] | 93 | 26 | 28.0% |  |  | 17 | 18.3% | 5 | 5.4% | 34 | 36.5% |  |  |  |  | 11 | 11.8% |  |  | 4 |
| Yang M, 2012 [[34](#_ENREF_34)] | 478 |  |  | 82 | 17.2% | 10 | 2.1% | 12 | 2.5% | 13 | 2.7% |  |  | 9 | 1.9% | 309 | 64.6% | 43 | 9.0% | 4 |
| Yang YC, 2011 [[35](#_ENREF_35)] | 1,798 | 67 | 3.7% | 462 | 25.7% | 482 | 26.8% | 148 | 8.3% | 245 | 13.6% | 2 | 0.1% | 91 | 5.1% | 245 | 13.6% | 56 | 3.1% | 5 |
| Yu J, 2011 [[36](#_ENREF_36)] | 239 | 9 | 3.8% | 169 | 70.7% | 27 | 11.3% | 8 | 3.3% |  |  |  |  |  |  | 16 | 6.7% | 10 | 4.2% | 4 |
| Zhan SW, 2008 [[30](#_ENREF_30)] | 93 | 16 | 17.2% | 48 | 51.6% | 18 | 19.4% |  |  | 3 | 3.2% |  |  | 8 | 8.6% |  |  |  |  | 4 |
| Zhang XH, 2007 [[37](#_ENREF_37)] | 38 |  |  | 31 | 81.6% |  |  |  |  |  |  |  |  | 6 | 15.8% | 1 | 2.6% |  |  | 4 |
| Zhang YP, 2009 [[38](#_ENREF_38)] | 162 | 26 | 16.0% | 47 | 29.0% | 29 | 17.9% | 29 | 17.9% |  |  |  |  |  |  | 31 | 19.2% |  |  | 4 |
| Zhang ZH, 2010 [[39](#_ENREF_39)] | 26 | 3 | 11.5% |  |  | 11 | 42.3% |  |  |  |  | 1 | 3.9% | 3 | 11.5% | 8 | 30.8% |  |  | 2 |
| Zhao LL, 2012 [[40](#_ENREF_40)] | 1024 | 356 | 34.8% |  |  | 86 | 8.4% | 186 | 18.2% | 264 | 25.8% |  |  |  |  | 132 | 12.9% |  |  | 3 |
| Zhao XC, 2010 [[41](#_ENREF_41)] | 225 | 57 | 25.3% | 47 | 20.9% | 22 | 9.8% | 28 | 12.4% | 31 | 13.8% |  |  | 20 | 8.9% | 19 | 8.4% | 1 | 0.4% | 3 |
| Zhao XH, 2009 [[42](#_ENREF_42)] | 402 | 108 | 26.9% |  |  | 138 | 34.3% | 25 | 6.2% | 90 | 22.4% |  |  |  |  |  |  | 41 | 10.2% | 4 |
| Zhu YH, 2012 [[43](#_ENREF_43)] | 66 |  |  | 34 | 51.5% |  |  | 3 | 4.5% |  |  |  |  | 19 | 28.8% |  |  | 10 | 15.2% | 4 |
| **Overall** | 20,873 | 1,591 | 7.6% | 4,632 | 22.2% | 3,977 | 19.1% | 1,316 | 6.3% | 2,762 | 13.2% | 18 | 0.1% | 1,311 | 6.3% | 2,580 | 12.4% | 2,685 | 12.9% |  |

**References**

1. Cao XY, Dai KT (2009) Reasons of 93 dropout cases in methadone maintenance treatment program. Practical Preventive Medicine 16: 1294-1295.

2. Chen A (2009) Predictors of retention related factors at the initial methadone maintenance treatment clinics in Guangdong province. Chinese Journal of Epidemiology 30: 1230-1233.

3. Chen C (2010) Analysis of hydrochloric acid Methadone Maintenance Treatment for Heroin Addicts. Chinese Community Doctors 12: 142-143.

4. Chen GH, Yang HT, Qian XC, Xu GY, Zhu YF, et al. (2008) Effectiveness evaluation of six-month community-based methadone maintenance treatment in Jiangsu Province. Chinese Journal of AIDS & STD: 590-593.

5. Chen W, Ling L, He q, Chen A, Chen J, et al. (2010) Performance evaluation and policy recommendations on community methadone maintenance treatment in Guangdong Province. Chinese Journal of Health Policy 3: 6.

6. Cheng XL, Xiao YK (2009) Analysis for the basic condition of the first clinics of Anhui methadone maintenance treatment. Anhui Medical Journal 30: 907-909.

7. Cui ZL, Wang C, Liu HJ, Ma CM, Dou ZJ, et al. (2012) Reason Analysis of the Dropout in the Process of 112 Cases on Methadone Maintenance Treatment in ZhengZhou City. Henan Journal of Preventive Medicine 23: 1-3.

8. Feng wd, Wei QH, Wei L, Bai Y, Shan GS (2007) Analysis of methadone maintenance treatment for heroin addicts in Liuzhou city. Journal of Public Health and Preventive Medicine 18: 31-33.

9. Hao C, Wu JL, Ruan YH, Yao HM, Yang XG, et al. (2007) Factors associated with retention in a community-based methadone maintenance treatment among heroin addicts. Zhonghua Yu Fang Yi Xue Za Zhi 41: 250-253.

10. He Q, Wang X, Xia Y, Mandel JS, Chen A, et al. (2011) New community-based methadone maintenance treatment programs in Guangdong, China, and their impact on patient quality of life. Subst Use Misuse 46: 749-757.

11. Huang YM, Tang ZZ, Liu W, Li BD, Lan GH, et al. (2012) Adherence rate and related factors of methadone maintenance treatment among heroin addicts in Lingshan, Guangxi. Chinese Journal of Disease Control & Prevention 16: 1036-1039.

12. Jia Y, Lu F, Zeng G, Sun X, Xiao Y, et al. (2008) Predictors of HIV infection and prevalence for syphilis infection among injection drug users in China: community-based surveys along major drug trafficking routes. Harm Reduct J 5: 29.

13. Jiang JH (2011) Analysis on the reasons among 290 patients who drop-out from the community-based drug maintenance and treatment clinics. Jiankang Bidu 11: 302-303.

14. Kong HQ (2011) Reasons for drop out of drug addicts in methadone maintenance treatment clinics. Chinese Journal of Aesthetic Medicine 20: 357.

15. Lai WS (2007) Reasons for heroin dependent patients’ dropout from methadone maintenance treatment. Chinese Journal of Drug Dependence 16: 299-301.

16. Li XM, Xiao LL, Gao SH, Jie CM, Huang Y (2011) Analysis on Reasons for Patient Drop-out from Methadone Maintenance Treatment in Hanchuan City. Preventive Medicine Tribune 17: 32-34.

17. Wang ZC, Zhong HJ, Wang L, Huang GY, Li SC, et al. (2007) Qualitative study of factors contributed to retention in a community-based methadone maintenance treatment among heroin users. Journal of Public Health and Preventive Medicine 18: 10-12.

18. Liu YJ, Deng PX, Xiong XY, Shuai YL, Wu W (2007) Effective evaluation on methadone maintenance treatment in Chaoyang District, Beijing. Chinese Journal of Drug Dependence 16: 302-306.

19. Lu ML, Li XG, Hu LY, Li F, Luo LH (2010) Influencing factors for dropout of 138 drug users on methadone maintenance treatment. Practical Preventive Medicine 17: 171-172.

20. Mu FK (2011) A study on 180 drug addicts on methadone maintenance treatment. China Medicine and Pharmacy 01: 117-118.

21. Pang L, Hao Y, Mi G, Wang C, Luo W, et al. (2007) Effectiveness of first eight methadone maintenance treatment clinics in China. AIDS 21 Suppl 8: S103-107.

22. Qian YH (2008) Effect of Methadone Maintenance Therapy on Drug Users in Wuxi. Occupation and Health 24: 450-452.

23. Shuai YL, Xiong XY, Sun Y, Sun LL (2010) Survey on reasons for drop out of methadone maintenance treatment in Beijing. Chinese Journal of Drug Dependence: 277-280.

24. Wang B, Zhao J, Qian XC, Pan XW, Gao Q, et al. (2010) Reasons and influencing factors of patient-loss in methadone maintenance treatment clinics in Wuxi. Zhonghua Liu Xing Bing Xue Za Zhi 31: 238-239.

25. Wang CQ, Liang QS, Xie HL, Su M, Su TX (2009) Study in compliance in methadone maintenance treatment. International Medicine & Health Guidance News 15: 78-80.

26. Wang P, Zhu JH, Yan J, Ou YS, Liu CF, et al. (2009) Analysis of patients' dropout from methadone maintenance treatment. Chinese Journal of Drug Abuse Prevention and Treatment 15: 77-80.

27. Wang YZ, Sun J (2008) Analysis of the efficacy of methadone maintenance treatment in 237 cases of heroin dependence patients. Practical Journal of Medicine & Pharmacy 25: 1304-1306.

28. Xing XZ, Du RR, Liu X, Xia Y (2012) An investigation and intervention on 177 patients dropout from methadone maintenance treatment. Chinese Journal of Drug Abuse Prevention and Treatment 18: 148-149.

29. Xu JS, Chen GH, Li L, Huan XP, Yang HT, et al. (2009) Retention and related factors of methadone treatment among drug users. Zhonghua Liu Xing Bing Xue Za Zhi 25: 779-780.

30. Xue LY, Pan QC, Zhu ZQ, Zhuang MH, Fang H, et al. (2008) Survey on reasons for drop out of methadone maintenance treatment in Shanghai. Chinese Journal of Drug Dependence 17: 192-196.

31. Yan JF, Zhang FY (2010) Study on the associated factors of compliance in methadone maintenance treatment. Journal of China Traditional Chinese Medicine Information 2: 17.

32. Yan H, Zhang R, Wang XP, Wang M, Liu H, et al. (2009) Analysis of reasons for patients leaving methadone maintenance treatment. Chinese Journal of Drug Dependence 18: 140-143.

33. Yang LS (2008) Reasons Dropout from Methadone Maintenance Treatment of 93 Cases. Journal of Dali University 7: 22-24.

34. Yang M, Yuan LH, Wu M, Xiao YG (2012) Survey Oil Patients Withdrawing from Methadone Maintenance Treatment in Luoxing District, Loudi City. Practical Preventive Medicine 19: 1582-1584.

35. Yang YC, Duan S, Xiang LF, Ye RH, Gao J, et al. (2011) Adherence and related determinants on methadone maintenance treatment among heroin addicts in Dehong prefecture, Yunnan province. Zhonghua Liu Xing Bing Xue Za Zhi 32: 125-129.

36. Yu J, Liu YXL, Xiao Qiong (2011) Reasons of drop-out in methadone maintenance and treatment clinics. Guide of China Medicine 9: 259-260.

37. Zhang XH (2007) Analysis of 117 cases on methadone maintenance treatment. Chinese Journal of Drug Dependence 16: 140-142.

38. Zhang YP, Tian Y, Fan JX, Xu YF (2009) Analysis of methadone maintenance treatment in Changchun city. Chinese Journal of Drug Abuse Prevention and Treatment 15: 316-319.

39. Zhang ZH, Yang NB (2010) Efficacy Evaluation of Methadone Maintenance Therapy on Drug Users in Huarong Country from 2008 to 2009. Practical Preventive Medicine 17: 1884-1886.

40. Zhao LL, Li XN, Wang MS, Lu HZ (2012) Reasons for drop out of heroin addicts in methadone maintenance treatment in Lanzhou. Chinese Journal of Drug Abuse Prevention and Treatment 21: 144-146.

41. Zhao XC, Zhang JM, Chen RZ (2010) Investigating the drop out reasons in methadone maintenance treatment. Journal of Applied Preventive Medicine 16: 235-236.

42. Zhao XH, K. LK, Liu DC, Yang HW, Sun HY (2009) On Reasons Lead to Missing in Maintenance Treatment. Journal of Preventive Medicine Information 25: 457-459.

43. Zhu YH, Wang X, Huang M (2012) Evaluation of effect of community-based methadone maintenance treatment for addicts. Journal of Jiujiang University (Natural Sciences) 27: 25-28.
