# Supplementary material for: Methadone Maintenance Treatment Participant Retention and Behavioural Effectiveness in China: A Systematic Review and Meta-Analysis
Source: PLoS One. 2013 Jul 26;8(7):e68906. doi: 10.1371/journal.pone.0068906 (PMC3724877; doi:10.1371/journal.pone.0068906)
Supplement: Table S3 — Remove this caption text. (DOCX) [file pone.0068906.s003.docx]

**Table S3. Percentage and likelihood of positive urine tests at baseline, 3, 6 and 12 months of follow-up.**

| **Study** | **Number of positive urine tests** | **Sample Size** | **Percentage of positive urine tests**  **(%, 95% CI)** | **Adjusted Odds Ratio**  **(95% CI)** |
| --- | --- | --- | --- | --- |
| Baseline |  |  |  |  |
| Deng HC, 2011 [[1](#_ENREF_1)] | 203 | 203 | 100.0 (100.0-100.0) |  |
| Duan YJ, 2008 [[2](#_ENREF_2)] | 99 | 99 | 100.0 (100.0-100.0) |  |
| Fu JK, 2010 [[3](#_ENREF_3)] | 52 | 80 | 65.0 (49.0-81.0) |  |
| Liu JK, 2009 [[4](#_ENREF_4)] | 112 | 112 | 100.0 (100.0-100.0) |  |
| Shi S, 2006 [[5](#_ENREF_5)] | 88 | 100 | 88.0 (74.9-99.9) |  |
| Shi S, 2008 [[6](#_ENREF_6)] | 301 | 329 | 91.5 (86.9-96.1) |  |
| *Subtotal* | 855 | 923 | 92.6 (90.1-95.2) | Ref |
| 3-month post MMT initiation | |  |  |  |
| Shi S, 2006 [[5](#_ENREF_5)] | 56 | 60 | 93.3 (83.7-100.0) |  |
| Shi S, 2008 [[6](#_ENREF_6)] | 181 | 329 | 55.0 (46.8-63.2) |  |
| *Subtotal* | 237 | 389 | 60.9 (53.5-68.3) | 0.47 (0.03-8.29) |
| 6-month post MMT initiation | |  |  |  |
| Deng HC, 2011 | 135 | 203 | 66.5 (56.6-76.4) |  |
| Fu JK, 2010 [[3](#_ENREF_3)] | 18 | 80 | 22.5 (8.5-36.5) |  |
| Shi S, 2006 [[5](#_ENREF_5)] | 11 | 40 | 27.5 (6.3-48.7) |  |
| *Subtotal* | 291 | 323 | 50.8 (42.4-59.1) | 0.05 (0.01-0.24) |
| 12-month post MMT initiation | |  |  |  |
| Duan YJ, 2008 [[2](#_ENREF_2)] | 23 | 99 | 23.2 (10.5-36.0) |  |
| Liu JK, 2009 [[4](#_ENREF_4)] | 29 | 112 | 25.9 (13.5-38.3) |  |
| *Subtotal* | 52 | 211 | 24.6 (15.7-33.5) | 0.002 (0.00-0.01) |

**References:**

1. Deng HC (2011) Analysis on 203 heroin addicts in community-based methadone maintenance treatment clinics for 180 days. Chinese Community Doctors 13: 97.

2. Duan YJ, Yin ZL, Xi CH, Li ZC, Gao Y, et al. (2008) Effect assessment of methadone maintenance treatment among heroin addicts in Ruili city. Chinese Journal of AIDS & STD 14: 240-242.

3. Fu JH, Hong LY, Li ZJ, Zhou XJ (2010) Effect of intervention among high-risk dropped out patients in methadone maintenance treatment. Jiangxi Medical Journal 45: 932-934.

4. Liu JK, Li LH, Chen YH, Liu D, Li L, et al. (2009) Evaluation of Methadone Maintenance Treatment for Heroin Users in Panzhihua. Journal of Preventive Medicine Information 25: 723-725.

5. Shi S, Huang YS, Huang HF, Li X, Li LP (2006) Associated factors for compliance to methadone maintenance treatment. Chinese Journal of Drug Dependence 15: 35-37.

6. Shi S, Huang YS, Li X, Zheng X, Huang HF, et al. (2008) Correlation factors influencing the effect of methadone maintenance treatment. Chinese Journal of Drug Dependence 17: 56-60.
