# Supplementary material for: Methadone Maintenance Treatment Participant Retention and Behavioural Effectiveness in China: A Systematic Review and Meta-Analysis
Source: PLoS One. 2013 Jul 26;8(7):e68906. doi: 10.1371/journal.pone.0068906 (PMC3724877; doi:10.1371/journal.pone.0068906)
Supplement: Table S4 — Remove this caption text. (DOCX) [file pone.0068906.s004.docx]

**Table S4. Summary of the demographic characteristics of the studies that reported the changes in risk behaviours.**

| **First author, published year** | **Language of Literature** | **Study Period** | **Study location** | **Provinces** | **Region** | **Type of Study** | **Method of sampling** | **Sampling size at recruitment** | **Age range (mean)** | **% of female** | **% of currently married** | **% of ethnic Han** | **Level of education (% Junior high and below )** | **Quality assessment score** |
| --- | --- | --- | --- | --- | --- | --- | --- | --- | --- | --- | --- | --- | --- | --- |
| Chen GH, 2008 [[1](#_ENREF_1)] | Chinese | 2006/02-2006/11 | Nanjing, Zhenjiang, Wuxi city | Jiangsu | East | Before-and-after | - | 554 | 13-52 (25.01) | 21.7% | 39.2% | 98.2% | 69.1% | 3 |
| Deng HC, 2011 [[2](#_ENREF_2)] | Chinese | - | Qujing city | Yunnan | Southwest | Before-and-after | - | 203 | 20-71 | 29.1% | - | - | 79.8% | 3 |
| Duan YJ, 2008 [[3](#_ENREF_3)] | Chinese | 2005/06-2006/12 | Ruili city | Yunnan | Southwest | Cohort | - | 99 | 20-55 (33.9) | 1.0% | 57.6% | 18.2% | 87.9% | 3 |
| Feng SQ, 2010 [[4](#_ENREF_4)] | Chinese | 2007/12-2008/12 | Changzhou city | Jiangsu | East | Before-and-after | - | 371 | N/A (34.23) | 21.0% | 43.1% | 99.2% | - | 4 |
| Fu JH, 2010 [[5](#_ENREF_5)] | Chinese | 2008/01-2008/06 | Nanchang city | Jiangxi | East | Before-and-after | - | 80 | N/A (37.6) | 22.5% | 28.8% | - | 68.8% | 4 |
| Fu LP, 2007 [[6](#_ENREF_6)] | Chinese | 2006/03-2006/09 | Ürümchi | Xinjiang | Northwest | Before-and-after | - | 958 | - | 11.8% | 42.8% | 31.2% | 68.0% | 3 |
| Huang YJ, 2012 [[7](#_ENREF_7)] | Chinese | 2009/10-2010/12 | Baise | Guangxi | South Central | Before-and-after | - | 150 | N/A (34.2) | 17.3% | 28.7% | 24.7% | 81.3% | 5 |
| Jiang A, 2009 [[8](#_ENREF_8)] | Chinese | 2006/10-2007/10 | Wuzhong city | Ningxia | Northwest | Before-and-after | - | 100 | 20-49 | 11.0% | - | 70.0% | 16.0% | 4 |
| Li XL, 2011 [[9](#_ENREF_9)] | Chinese | 2005/09-2007/04 | Hengyang, Yueyang, Changsha, Hongjiang, Chenzhou, Qidong, Shaodong | Hunan | South Central | Before-and-after | - | 705 | - | 15.0% | 15.0% | N/A | N/A | 3 |
| Liu JK, 2009 [[10](#_ENREF_10)] | Chinese | 2005/06-2006/12 | Panzhihua city | Sichuan | Southwest | Cohort | - | 112 | 20-60 (34.8) | 23.2% | 51.8% | 87.5% | 85.7% | 3 |
| Liu WY, 2012 [[11](#_ENREF_11)] | Chinese | 2009/01-2010/06 | Chongqing | Sichuan | Southwest | Before-and-after | - | 650 | 18-59 (38.1) | 30.2% | 53.4% | 99.4% | 67.1% | 5 |
| Liu YJ, 2007 [[12](#_ENREF_12)] | Chinese | 2005/08-2007/02 | Chaoyang District | Beijing | North | Before-and-after | - | 130 | 22-54 (38.34) | 20.0% | 50.8% | 91.5% | 50.8% | 3 |
| Long ZY, 2006 [[13](#_ENREF_13)] | Chinese | 2004/06-2005/06 | Guiyang city | Guizhou | South Central | Before-and-after | - | 538 | - | 27.1% | 21.4% | 95.2% | 55.2% | 3 |
| Pang L, 2007 [[14](#_ENREF_14)] | English | 2004/04-2005/11 | N/A | Sichuan, Yunnan, Guizhou, Guangxi, Zhejiang | N/A | Before-and-after | - | 1662 | - | 24.9% | - | 91.0% | 69.8% | 5 |
| Qian YH, 2008 [[15](#_ENREF_15)] | Chinese | 2006/03-2006/10 | Wuxi city | Jiangsu | East | Before-and-after | - | 965 | - | 21.0% | 47.1% | - | 71.9% | 4 |
| Qu BW, 2009 [[16](#_ENREF_16)] | Chinese | 2006/09-2007/10 | Jiangmen city | Guangdong | South Central | Before-and-after | - | 80 | 21-50 | 10.0% | - | 97.5% | 73.8% | 6 |
| Shi S, 2006 [[17](#_ENREF_17)] | Chinese | 2004/06-2005/03 | Nanning city | Guangxi | South Central | Before-and-after | - | 100 | - | 0.8% | 38.0% | - | 65.0% | 4 |
| Shi S, 2008 [[18](#_ENREF_18)] | Chinese | 2004/06-2006/07 | Nanning city | Guangxi | South Central | Before-and-after | - | 329 | - | 19.8% | 50.2% | - | 55.6% | 4 |
| Sun QY, 2011 [[19](#_ENREF_19)] | Chinese | 2008/11-2009/12 | Lianyungang | Jiangsu | East | Before-and-after | - | 207 | 18-53 (31.67) | 13.0% | 45.4% | N/A | 52.7% | 5 |
| Tang RH, 2012 [[20](#_ENREF_20)] | Chinese | 2005/06-2011/04 | Dehong | Yunnan | Southwest | Before-and-after | - | 2562 | N/A (33.0) | 3.9% | 57.8% | 41.5% | 86.5% | 6 |
| Tang XY, 2008 [[21](#_ENREF_21)] | Chinese | 2006/01 | Beihu District | Hunan | South Central | Before-and-after | - | 196 | 23-47 (34.75) | 24.5% | - | - | - | 3 |
| Wei XL, 2008 [[22](#_ENREF_22)] | Chinese | 2005/10-2007/09 | Xi'an city | Shaanxi | Northwest | Before-and-after | Random sampling | 972 | N/A (37.08) | 16.0% | 51.0% | 90.7% | 63.3% | 5 |
| Xue LY, 2006 [[23](#_ENREF_23)] | Chinese | 2005/05-2006/06 | Changning District | Shanghai | East | Before-and-after | - | 115 | 23-55 | 16.7% | 33.9% | - | 67.0% | 4 |
| Zhang HF, 2009 [[24](#_ENREF_24)] | Chinese | 2006/09-2008/03 | Hanzhong city | Shaanxi | Northwest | Before-and-after | - | 120 | 20-54 (32.18) | 16.7% | 40.0% | 98.3% | 95.0% | 4 |
| Zhang ZH, 2010 [[25](#_ENREF_25)] | Chinese | 2008/12-2009/12 | Huarong County | Hunan | South Central | Before-and-after | - | 220 | - | 15.5% | 46.4% | - | 28.6% | 2 |
| Zhao YT, 2009 [[26](#_ENREF_26)] | Chinese | 2006 | Guangzhou city | Guangdong | South Central | Before-and-after | - | 65 | N/A (36.75) | 6.2% | - | 100.0% | 78.5% | 6 |
| Zheng WX, 2012 [[27](#_ENREF_27)] | Chinese | 2006/10-2007/12 | N/A | Fujian | East | Before-and-after | - | 587 | N/A (33.8) | 13.6% | 44.6% | 97.9% | 81.3% | 4 |
| Zhu YH, 2012 [[28](#_ENREF_28)] | Chinese | 2009/03-2010/12 | Jiujiang | Jiangxi | East | Before-and-after | - | 342 | 21-56 (36) | 14.0% | N/A | N/A | 83.3% | 4 |

**References**

1. Chen GH, Yang HT, Qian XC, Xu GY, Zhu YF, et al. (2008) Effectiveness evaluation of six-month community-based methadone maintenance treatment in Jiangsu Province. Chinese Journal of AIDS & STD: 590-593.

2. Deng HC (2011) Analysis on 203 heroin addicts in community-based methadone maintenance treatment clinics for 180 days. Chinese Community Doctors 13: 97.

3. Duan YJ, Yin ZL, Xi CH, Li ZC, Gao Y, et al. (2008) Effect assessment of methadone maintenance treatment among heroin addicts in Ruili city. Chinese Journal of AIDS & STD 14: 240-242.

4. Feng SQ, Zhou JB, Guo YL, Shi TP, Li JH, et al. (2010) Effective evaluation of community-based methadone maintenance treatment for heroin addicts. Zhonghua Liu Xing Bing Xue Za Zhi 26: 924-925.

5. Fu JH, Hong LY, Li ZJ, Zhou XJ (2010) Effect of intervention among high-risk dropped out patients in methadone maintenance treatment. Jiangxi Medical Journal 45: 932-934.

6. Fu LP, Li F, Zhang ZZ, Mo LR (2007) Effective Evaluation on Parts of Community-based Methadone Maintenance Treatment Clinics for Heroin Addicts in Xinjiang. Endemic Diseases Bulletin 22: 17-19.

7. Huang YJ, Huang JG, Lv K, Zhou MX, Xia CL, et al. (2012) An outcome assessment on methadone maintenance treatment in Youjiang district, Baise city. Journal of Youjiang Medical University for Nationalities 34: 461-463.

8. Jiang A, Zhao JH, Wang XZ, Han QQ, Chen SG, et al. (2009) Effect analysis of the heroin addicts with methadone maintenance treatment in experimental units of Wuzhong in Ningxia. Modern Preventive Medicine 36: 2920-2922.

9. Li XL, Tan HZ, Ou QY, Chen MS, Zhang H (2011) Efficacy of methadone maintenance treatment among HIV positive and HIV negative heroin addicts. Chinese Journal of Drug Dependence 20: 362-366.

10. Liu JK, Li LH, Chen YH, Liu D, Li L, et al. (2009) Evaluation of Methadone Maintenance Treatment for Heroin Users in Panzhihua. Journal of Preventive Medicine Information 25: 723-725.

11. Liu WY, Wang S, Gong B, Luo HZ, Chen YY, et al. (2012) Effective Evaluation of Methadone Maintenance Treatment Combined with Comprehensive Intervention for Six Months. Acta Medicinae Universitatis Scientiae et Technologiae Huazhong 41: 190-194.

12. Liu YJ, Deng PX, Xiong XY, Shuai YL, Wu W (2007) Effective evaluation on methadone maintenance treatment in Chaoyang District, Beijing. Chinese Journal of Drug Dependence 16: 302-306.

13. Long ZY, Wu ZY, Du B, Wang L, Guo GZ, et al. (2006) Situation of 538 heroin addicts undertaking methadone maintenance treatment. Chinese Journal of Drug Dependence 15: 38-40.

14. Pang L, Hao Y, Mi G, Wang C, Luo W, et al. (2007) Effectiveness of first eight methadone maintenance treatment clinics in China. AIDS 21 Suppl 8: S103-107.

15. Qian YH (2008) Effect of Methadone Maintenance Therapy on Drug Users in Wuxi. Occupation and Health 24: 450-452.

16. Ou BW, Gao XX, Huang MJ, Liang YL, Hu YL (2009) Analysis of the effects of methadone maintenance therapy among heroin-addicts in Jiangmen urban. Modern Hospital 9: 142-143.

17. Shi S, Huang YS, Huang HF, Li X, Li LP (2006) Associated factors for compliance to methadone maintenance treatment. Chinese Journal of Drug Dependence 15: 35-37.

18. Shi S, Huang YS, Li X, Zheng X, Huang HF, et al. (2008) Correlation factors influencing the effect of methadone maintenance treatment. Chinese Journal of Drug Dependence 17: 56-60.

19. Sun QY, Zhang YF, Zhang J, Xu M, Chen GH (2011) Evaluation of the community methadone maintenance treatment in Lianyungang city. Acta Universitatis Medicinalis Nanjing (Natural Science) 31: 1471-1475.

20. Tang RH, Duan S, Yang YC, Xiang LF, Ye RH, et al. (2012) Analysis on social effects of methadone maintenance treatment in Dehong Prefecture, Yunnan Province. Chinese Journal of Disease Control & Prevention 16: 1044-1048.

21. Tang XY, Hou SQ, Tang JH (2008) Hunan Beihu methadone maintenance treatment in socio-economic evaluation. Chinese Journal of Drug Dependence 17: 380-382.

22. Wei XL, Li HX, Ma CF, Liu JF (2008) Effective evaluation of methadone maintenance treatment for heroin dependent patients in Xi'an city. Chinese Journal of Drug Dependence 17: 197-201.

23. Xue LY, Xu CL, Pan QC, Zhang MH, Bi H, et al. (2006) Evaluation of the Therapeutic Effect of Methadone Maintenance in 115 Cases of Heroin Addicts. Chinese Journal of Drug Abuse Prevention and Treatment 12: 255-257.

24. Zhang HF, Deng KW, Zhang YF, Tong HY, Long HY (2009) Efficacy evaluation on methadone maintenance treatment in Hanzhong, Shaanxi. Chinese Journal of Drug Dependence 18: 43-46.

25. Zhang ZH, Yang NB (2010) Efficacy Evaluation of Methadone Maintenance Therapy on Drug Users in Huarong Country from 2008 to 2009. Practical Preventive Medicine 17: 1884-1886.

26. Zhao YT, Xu HF, Fan LR (2009) Evaluation for the Community-Based Methadone Maintenance Treatment in Guangzhou City. Journal of Tropical Medicine 9: 329-331.

27. Zheng WX, Chen G (2012) Evaluation on methadone maintenance treatment in Fujian province. Strait Journal of Preventive Medicine 18: 72-74.

28. Zhu YH, Wang X, Huang M (2012) Evaluation of effect of community-based methadone maintenance treatment for addicts. Journal of Jiujiang University (Natural Sciences) 27: 25-28.
