# Supplementary material for: Methadone Maintenance Treatment Participant Retention and Behavioural Effectiveness in China: A Systematic Review and Meta-Analysis
Source: PLoS One. 2013 Jul 26;8(7):e68906. doi: 10.1371/journal.pone.0068906 (PMC3724877; doi:10.1371/journal.pone.0068906)
Supplement: Figure S1 — Remove this caption text. (DOCX) [file pone.0068906.s005.docx]

**Figure S1. Flow chart of study selection for retention among MMT participants.**
