# Supplementary material for: Methadone Maintenance Treatment Participant Retention and Behavioural Effectiveness in China: A Systematic Review and Meta-Analysis
Source: PLoS One. 2013 Jul 26;8(7):e68906. doi: 10.1371/journal.pone.0068906 (PMC3724877; doi:10.1371/journal.pone.0068906)
Supplement: Figure S3 — (a) Percentage of MMT participants who had injected drugs in the past one month. (b) Percentage of MMT participants who had shared syringe in the past one month. (c) Percentage of MMT participants who had consistent condom use during any sexual intercourse in the past one month. (d) Percentage of MMT participants who sell sex for drug in the past three months. (DOCX) [file pone.0068906.s007.docx]

**Figure S3. Changes in risk injecting and sexual behaviours among retained MMT participants.**

**(a) Percentage of MMT participants who had injected drugs in the past one month.**

**(b) Percentage of MMT participants who had shared syringe in the past one month.**

**(c) Percentage of MMT participants who had consistent condom use during any sexual intercourse in the past one month.**

**(d) Percentage of MMT participants who sell sex for drug in the past three months.**
