# Supplementary material for: Methadone Maintenance Treatment Participant Retention and Behavioural Effectiveness in China: A Systematic Review and Meta-Analysis
Source: PLoS One. 2013 Jul 26;8(7):e68906. doi: 10.1371/journal.pone.0068906 (PMC3724877; doi:10.1371/journal.pone.0068906)
Supplement: Text S1 — (DOCX) [file pone.0068906.s009.docx]

**Text S1. Search strategy**

Keywords used in the database search for (1) included (‘Methadone’ [MeSH] *OR* ‘Methadone Maintenance Treatment’ *OR* ‘Methadone Maintenance Therapy’ *OR* ‘Methadone Maintenance’) *AND* (‘retention’ [MeSH] *OR* ‘dropped out’) *AND* (‘China’ *OR* ‘China Mainland”); while key words used in the database search for (2) included (‘Methadone’ [MeSH] *OR* ‘Methadone Maintenance Treatment’ *OR* ‘Methadone maintenance treatment’ *OR* ‘Methadone Maintenance’) *AND* (‘behaviours‘ *OR* ‘sexual behaviours” *OR* ‘drug behaviours” *OR* ‘injecting‘ *OR* ‘sharing’ *OR* ‘condom use’) *AND* (‘China‘ *OR* ‘China Mainland’). We also performed a manual search of the bibliographies of published full text articles.
